# The Italian neuromuscular registry: a coordinated platform where patient organizations and clinicians collaborate for data collection and multiple usage

**DOI:** 10.1186/s13023-018-0918-z

**Published:** 2018-10-04

**Authors:** Anna Ambrosini, Daniela Calabrese, Francesco Maria Avato, Felice Catania, Guido Cavaletti, Maria Carmela Pera, Antonio Toscano, Giuseppe Vita, Lucia Monaco, Davide Pareyson

**Affiliations:** 1 Fondazione Telethon, Via Poerio 14, 20129 Milan, Italy; 2 UOC Malattie neurodegenerative e neurometaboliche rare, Fondazione IRCCS Istituto Neurologico Carlo Besta, Milan, Italy; 3 University of Ferrara, Ferrara, Italy; 4 Astir s.r.l., Milan, Italy; 5 School of Medicine and Surgery and Experimental Neurology Unit, University of Milano-Bicocca, Monza, Italy; 6 Paediatric Neurology and Centro Clinico Nemo, Catholic University and Policlinico Gemelli, Rome, Italy; 7 Department of Clinical and Experimental Medicine, University of Messina, Messina, Italy

**Keywords:** Neuromuscular disorders, Clinical network, Patient registries, Patient engagement

## Abstract

**Background:**

The worldwide landscape of patient registries in the neuromuscular disease (NMD) field has significantly changed in the last 10 years, with the international TREAT-NMD network acting as strong driver. At the same time, the European Medicines Agency and the large federations of rare disease patient organizations (POs), such as EURORDIS, contributed to a great cultural change, by promoting a paradigm shift from product-registries to patient-centred registries. In Italy, several NMD POs and Fondazione Telethon undertook the development of a TREAT-NMD linked patient registry in 2009, with the referring clinical network providing input and support to this initiative through the years. This article describes the outcome of this joint effort and shares the experience gained.

**Methods:**

The Italian NMD registry is based on an informatics technology platform, structured according to the most rigorous legal national and European requirements for management of patient sensitive data. A user-friendly web interface allows both direct patients and clinicians’ participation. The platform’s design permits expansion to incorporate new modules and new registries, and is suitable of interoperability with other international efforts.

**Results:**

When the Italian NMD Registry was initiated, an ad hoc legal entity (NMD Registry Association) was devised to manage registries’ data. Currently, several disease-specific databases are hosted on the platform. They collect molecular and clinical details of individuals affected by Duchenne or Becker muscular dystrophy, Charcot-Marie-Tooth disease, transthyretin type-familial amyloidotic polyneuropathy, muscle glycogen storage disorders, spinal and bulbar muscular atrophy, and spinal muscular atrophy. These disease-specific registries are at different stage of development, and the NMD Registry itself has gone through several implementation steps to fulfil different technical and governance needs. The new governance model is based on the agreement between the NMD Registry Association and the professional societies representing the Italian NMD clinical network. Overall, up to now the NMD registry has collected data on more than 2000 individuals living with a NMD condition.

**Conclusions:**

The Italian NMD Registry is a flexible platform that manages several condition-specific databases and is suitable to upgrade. All stakeholders participate in its management, with clear roles and responsibilities. This governance model has been key to its success. In fact, it favored patient empowerment and their direct participation in research, while also engaging the expert clinicians of the Italian network in the collection of accurate clinical data according to the best clinical practices.

**Electronic supplementary material:**

The online version of this article (10.1186/s13023-018-0918-z) contains supplementary material, which is available to authorized users.

## Background

Neuromuscular diseases (NMDs) include a wide range of pathological rare conditions, mainly of genetic origin, that affect muscles and motor or sensory neurons [1–3].

Due to the high variability of genetic defects and clinical phenotypes, the development of therapeutic approaches has always been very challenging. This prompted the NMD scientific community and patient organizations (POs) to network in order to address together the main bottlenecks and translational challenges. In 2007, the TREAT-NMD project started as a European Committee (EC) funded “Network of excellence” with the purpose of supporting translational research in the NMD field and promote trial readiness [4]. This was particularly urgent for the two most common paediatric diseases, Duchenne muscular dystrophy (DMD) and spinal muscular atrophy (SMA), for which clinical trials were approaching [5]. A major recognised need was to collect, in an organized way, useful information on patients to support feasibility analyses and clinical trial design, and to favour implementation of clinical trials with the rapid identification of the most suitable patients. Therefore, TREAT-NMD engaged all partners in the development of DMD and SMA patient registries linked to its international Global Registry, which is part of a research infrastructure acknowledged as an “IRDiRC (International Rare Diseases Research Consortium) recognised resources” [6].

Fondazione Telethon (Telethon) was the Italian partner of the TREAT-NMD EC project and, in this role, it engaged the Italian neuromuscular POs to develop NMD patient registries. A legal entity called “Associazione del Registro dei pazienti con malattie neuromuscolari” (ADR; NMD Patients Registry Association) was established in 2009 to manage the activity and to guarantee proper data stewardship to all stakeholders [7]. Current members of the ADR are the following Italian POs: ACMT-Rete (the main organisation of patients with CMT), AISLA (the main organisation of patients with amyotrophic lateral sclerosis), ASAMSI and Famiglie SMA (the two main organisations of patients with SMA), UILDM (the umbrella organization of patients with muscular dystrophies or other NMD) and Telethon.

### The Italian NMD clinical network

Clinical researchers working in the Italian NMD centres involved in NMD diagnosis and care belong to the scientific associations for the study of muscle (Associazione Italiana Miologia = AIM, Italian Myology Association) and peripheral nerve (Associazione italiana per lo studio del Sistema Nervoso Periferico = ASNP, Italian Peripheral Nerve Association) diseases. During the last couple of decades, AIM and ASNP were fundamental in building the Italian NMD clinical network and promoting many collaborative studies, which involved the majority of the Italian tertiary clinics. Telethon was also instrumental in this networking activity by providing support to several multicentre studies including the development of clinical registries [8–14].

In 2015, AIM, ASNP and Telethon signed a Memorandum of Understanding to start a new initiative called “NMD Alliance”, with the purpose of advancing NMD clinical research in Italy. One of the main goals of the NMD Alliance is to consolidate and expand the Italian NMD Registry by incorporating new disease registries and map the activities of the clinical centres. To address this objective, in 2017 the NMD Alliance and ADR co-signed an “Agreement on Registry governance and data stewardship” (Agreement). The Agreement is an important step forward that codifies the relationship among all stakeholders who interact with the NMD Registry. In fact, clear and transparent management roles, as well as inclusion of all stakeholders in the process are recognised as key factors for the success of patient registries, as they favour the endorsement of the initiative and help addressing the many hurdles and complexities [15].

### Patient-driven and clinician-driven registries

The interest for rare disease patient registries has enormously grown over the last years. All stakeholders acknowledge that well-structured databases are tools of great scientific and practical value to improve patient care [16–21]. High quality data may enable studies on disease epidemiology, natural history, functional outcomes or real world evaluation of drug efficacy and post-marketing drug surveillance. Moreover, registries can facilitate feasibility studies, trial design and rapid identification of the most suitable patients to be included in clinical trials. Depending on the purpose, a registry may collect data directly from patients and their caregivers, or require the involvement of experts in the disease. In any case, in order to fulfil expectations, data need to be of the highest quality, validated and maintained up-to-date [19, 20].

In line with the TREAT-NMD priorities, the first two registries developed by ADR addressed DMD and SMA and were, consequently, linked to the TREAT-NMD Global Registry; they envisage the direct involvement of patients, or parents in case of children, in the data collection. The presence of strong Italian POs focused on these diseases facilitated such implementation. Another DMD registry linked to TREAT-NMD is operating in Italy, managed by the Italian Parent Project PO; discussion is ongoing on how to merge the two DMD registries.

In addition, ADR acknowledged the need expressed by the clinicians of the Italian NMD network to develop clinician-driven registries for groups of disorders for which treatments were available or in the therapeutic pipeline and offered the opportunity to host these databases on the NMD Registry platform. Therefore, several clinician-driven registries were deployed during the last 5 years, namely for Charcot-Marie-Tooth disease (CMT), muscle glycogen storage disorders (MGSD), spinal and bulbar muscular atrophy (SBMA), and transthyretin type-familial amyloidotic polyneuropathy (TTR-FAP). Purpose and structure of these new registries fit with the definition given by Gliklich and colleagues of a registry as “an organized system that uses observational study methods to collect uniform data (clinical and other) to evaluate specified outcomes for a population defined by a particular disease, condition, or exposure, and that serves one or more predetermined scientific, clinical, or policy purposes” [15].

Overall, the Italian NMD Registry is an established initiative for data collection through a flexible technical tool; it is managed by a legal entity with transparent procedures and is involving all key stakeholders. Purpose of this article is to describe its structure and unique governance features, highlight the scopes of the disease-specific registries with reference to the international context, and provide a critical analysis of the experience gained so far.

## Methods

### IT data management platform

The informatics technology (IT) platform is a software infrastructure that provides basic functionalities such as: disease-specific forms for personal and clinical data collections (connected via pseudonymised codes); a module for the registry configuration of the participating clinical centres and their staff; a module for operator authentication; safety and protection solutions aligned with local regulations; storage solutions and data recovery. The IT service provider is Astir s.r.l. [22], which works in close collaboration with the ADR and with clinicians to design the best possible structure to fulfil clinicians’ needs, and is now aligning with the RD-Connect experts to adopt the international standards for interoperability. The procedure for data collection is compliant with the Italian Policy on Privacy for the management of sensitive data (the former Legislative Decree no. 196/2003 and the more recent EU General Data Protection Regulation, GDPR2016/279) [23]. Essentially, the following measures are in place: a) security and multilevel access for patients, specialists and operators; b) patient’s personal information stored in a separated compartment, with automatically-generated pseudonymisation codes; c) data encryption applied at different levels; d) software application and database (data storage) hosted in dedicated virtual servers provided by a Cloud Service.

### Modules

The NMD Registry is customisable and its design allows development by modules. Some of these are implemented on different registries having common features, while others are registry-specific minimal datasets. New forms, dedicated for instance to Patient Reported Outcomes (PRO), surveys or clinical study protocols, can be connected to the same patient’s pseudonymisation code.

### Intranet system

The IT platform collects information on the clinical investigators involved in each registry. Each expert accesses the system with personal codes and according to his/her role (Scientific Coordinator, Curator, other participant responsible for data entry or data validation). In addition to managing the individual access to the databases, the IT system can also function as a secure intranet system for data sharing, to support for instance diagnosis consultation, make standards of care guidelines available, and, in general, promote secure communication among clinical centres and collect information about their NMD expertise and activities.

### Web portal

Secure access to the NMD Registry by individuals affected by the disease or clinicians that participate in data collection occurs through a web portal [7]*.* This website also provides information on the governance model, illustrates the main goals of the NMD Registry and of each specific disease registry. In particular, a web page specific for each registry illustrates the type of data that are collected, the composition of the Scientific Committee, and the operating procedures, with a link to the information leaflet and Patient Consent form. When interested individuals create their account, they must consent on privacy policy and accept the online Patient Consent form. The registrants complete the process by signing the paper version of the Informed Consent and making it available to the Registry Curator or the referring clinical centre.

### Standard operating procedures

The NMD Registry has developed Standard Operating Procedures (SOPs), addressing (among others): how to create a new registry, role and responsibilities of all actors, modalities of data registration (clinical reported forms and support by clinicians to patients for the patients’ reported forms), and data access.

## Results

### The governance

#### The ADR legal entity

**Fig. 1:**
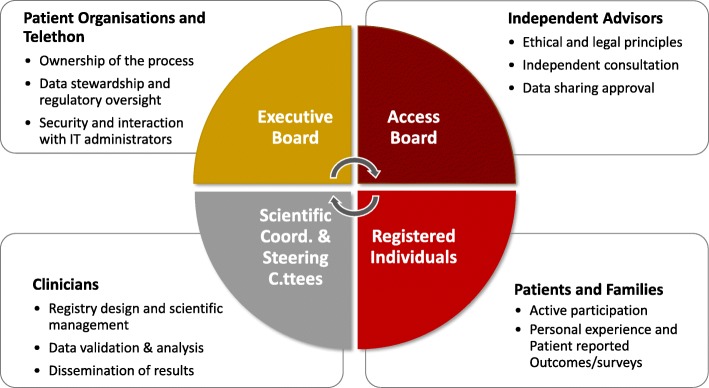
Governance framework of the NMD Registry. The picture illustrates the groups of stakeholder that participate in the NMD registry and their specific roles and contribution

The Italian NMD Registry is under the responsibility of a dedicated legal entity (the ADR)*.* A charter regulates its activities. The Italian NMD Registry is recorded in the Italian Data Protection Authority Register and is compliant with the Italian and European directives on data protection (EU GDPR 2016/279). The main governing body is the Executive Board, which includes a representative from each PO and Telethon; the President is always a PO representative, with a two-year mandate. When a new registry joins the NMD Registry, the referring Italian POs are invited to become members of the ADR and a representative joins the Executive Board. This has been the case for ACMT-Rete and this activity is now in progress for MGSD and TTR-FAP POs (Fig. 1).

#### The ethics and legal advisory council (ELAC)

ELAC is an independent board composed by five experts in ethical and legal issues, who advice the ADR on these matters. At the start of the initiative, the ELAC provided ADR with advice on the Italian and international legal requirements on data protection, validated the privacy design of the IT platform, the data management procedures and flowchart, and approved the Italian version of the Informed Consent for DMD and SMA registries. The ELAC also provided the Scientific Coordinator of the CMT Registry with advice on the Clinical Protocol and the patient Informed Consent before the submission of the documents for approval to the Institutional Review Board (IRB) of the coordinator and partners’ centres. The CMT registry was the first clinician-reported database hosted on the platform and the procedure developed for its implementation paved the way for the other disease-specific registries that followed. The ELAC also acted as independent consulting body for the evaluation of each request of data access addressed directly to the Italian NMD Registry. As described more in detail below, the ELAC model is now evolving into a new council defined “Access Board”.

#### The scientific steering committees

**Fig. 2:**
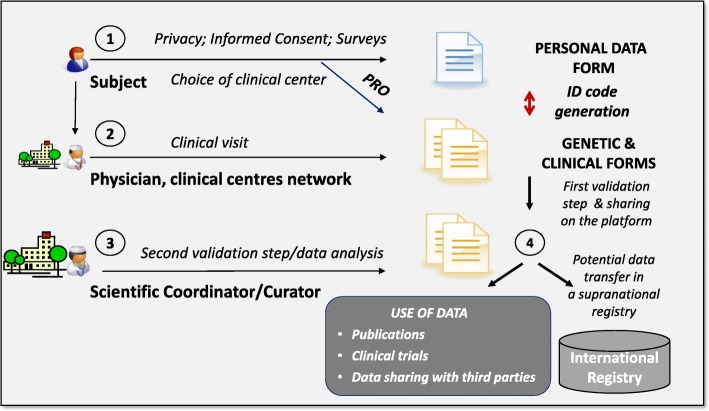
Standard Operating Procedures (SOPs) of the NMD Registry. 1. Individuals living with a NMD condition register their personal data and may fill in dedicated surveys or Patient Reported Outcomes (PROs). Filling in the personal data triggers the generation of a pseudonimysation code (ID), which will be attached to any data thereafter collected. Subjects select a preferred clinical centre if required by the specific registry. 2. Physicians collect and validate the medical data. 3. The Scientific Coordinator/Curator supervise the registry activity, and access data for final validation and data analysis. 4. Policies are in place for data usage and sharing

A major implementation of the NMD Registry concerned the inclusion of registries collecting clinician-reported items (Table 1, Fig. 2). These registries, promoted by groups of interest from the Italian NMD clinical network, started as databases of observational studies with the explicit intention to maintain and re-use data as proper registries. Their scientific aims entail the collection of natural history data, validation of functional measures, studies on quality of life and care management, monitoring efficacy of available treatments in the real world (where applicable).

**Table 1:** Disease-specific databases hosted on the NMD Registry platform

Registry	No. registered individuals (June 2018)	No. of clinical centres	Reference model	Items	Registration manner
Duchenne and Becker muscular dystrophy (DMD/BMD)	161	1 (Curator)	TREAT-NMD [4]	General clinical features	Patient reported
Spinal muscular atrophy (SMA)	560	1 (Curator)	TREAT-NMD [4]	General clinical features, treatments	Patient reported
Charcot-Marie-Tooth disease (CMT) -------------------- CMT surveys	721 -------------------- 306	9	Inherited Neuropathy Consortium (INC) of the Rare Disease Research Network, (NCATS, NIH) [32, 33] --------------------	Natural history, outcome measures ------------------- Patient reported outcomes	Dual reported ------------- Patient reported
Muscle glycogenoses (MGSD)	260 *(foreseen 500)*	*13*	European Neuromuscular Centre (ENMC) consensus [41], Euromac network [42]	Natural history, outcome measures	Clinician reported
Spinal & bulbar muscular atrophy (SBMA)	119 *(foreseen 200)*	*4*	ENMC consensus [12, 37]	Natural history, outcome measures	Dual reported
Transthyretin type-Familial Amyloidotic Polyneuropathy (TTR – FAP)	206 *(foreseen 270 + 100 carrier)*	*10*	European TTR-FAP Network (ATTReuNET) [44]	Natural history, outcome measures, treatments	Dual reported

ADR made the IT platform available to these registry-based observational studies, with the scope of favouring the long-term maintenance, update and re-use of data. Dedicated Steering Committees, including the Scientific Coordinator, the principal investigator of each partner centre and other experts, are responsible for the strategic development and management of each registry. The IRBs of all clinical centres involved in data collection approved the protocols and the Informed Consent forms.

The increased complexity of the NMD Registry platform prompted the ADR Executive Board to revise the governance process, in order to include the different stakeholders’ roles and views. In the new management scheme, the Scientific Coordinators participate in the governance on behalf of their Steering Committees, and interact with the Executive Board (Fig. 1).

#### The ADR-NMD Alliance agreement

The Agreement describes the roles of POs, Telethon, AIM and ASNP, and of the independent experts that participate in the Access Board. It also illustrates the general principles that regulate the different phases of a registry’s life, namely: i) development of new registries; ii) management and use of data by participating centres (Coordinator and Steering Committee’s clinical members); iii) access policies by third parties (clinical researchers or other professionals who have interest in consulting the data and Industry); iv) dissemination of results; v) sustainability. Specific details on the registry management, which can slightly vary from one disease-specific registry to another, are then addressed in the respective protocols and are reflected in the legal contracts for data management that are co-signed by ADR and each participating centre.

The NMD Alliance may facilitate the establishment of a new registry; moreover, it supports the ADR in the definition of its strategic vision, in order to keep up with clinical research innovation in the NMD field.

#### Data sharing

When a request for consultation of the Italian DMD and SMA registries affiliated to TREAT-NMD is channelled through the international TREAT-NMD network, ADR relies on approval process of the International TREAT-NMD Global Database Oversight Committee [4, 24, 25], of which it is a member.

Up to now, for any other request that addresses specifically the Italian NMD Registry, the ADR asked ethical and legal advice to its ELAC. The increased complexity of the NMD Registry platform, however, meant that this model was no longer adequate and a new consultative board named “Access Board” is now replacing the ELAC, which includes proper expertise to better address the scientific issues that may arise regarding any enquiries. Therefore, the main remit of the Access Board is to evaluate any external requests, relying on the ethical, legal or scientific competences, and independence of its members. To avoid conflicts of interest, the clinical experts that are members of the Access Board should not participate in any disease-specific registry hosted on the NMD registry platform. In case of requests by third parties, both the Scientific Coordinator of the targeted registry and the referring PO’s representative participate in the discussion as informed persons, without voting rights. This model is the outcome of in depth discussion between ADR and the NMD Alliance, to which also the former ELAC members took part by helping ADR and clinicians to define clear roles for all stakeholders and, ultimately, designing a rigorous and transparent process that safeguards all rights.

#### Funding & sustainability

The establishment of the ADR legal entity entailed also the creation of a fund with equal contribution by all members. This fund was initially invested in the development of the IT structure according to the legal and technical requirements, the design of the web portal, and the creation of the first three disease-specific databases. The business plans of the registries developed more recently (muscle glycogenoses, SBMA, and TTR-FAP) were supported by a grant awarded to the Scientific Coordinators and partners by Telethon within a specific competitive call dedicated to NMD clinical studies. The allocated budget concerns the development of the database and part-time support to personnel engaged in data collection and processing, from data entry to validation. The clinical Host Institutions and the National Health System support the work carried out by clinicians to perform molecular diagnosis analyses and visit patients for the accurate data collection.

Telethon is now directly supporting costs for IT maintenance and general management activities. Alternative or complementary sustainability opportunities are being sought.

### The registries

#### General features

All databases entail that affected individuals, or their parents/legal guardians in case of minor or incapacitated subjects, start the registration process by creating their own account.

**Fig. 3:**
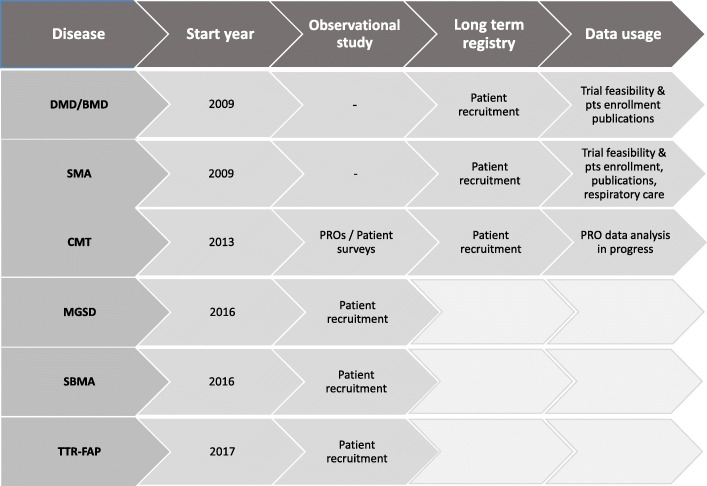
Registry implementation steps. DMD and SMA registries did not undergo an observational study stage. The CMT registry has had two components, a long term registry database (ongoing) and a focused observational study based on Patient Reported Outcomes (PROs) and surveys (2014–2017). The MGSD, SBMA and TTR-FAP registries have been developed as observational studies and are now progressing into the stage of long-term registries

The IT platform hosts several databases with patient- and/or clinician-reported forms at different level of implementation (Table 1 and Fig. 3). The DMD and SMA registries are based on patient-reported forms. All other registries implemented a dual registration mode, with patients starting the process by filling in their personal data and indicating an expert clinician within a given list, who will record their clinical data collected through an accurate visit. In addition, patients may contribute their own information on the disease progression or daily life experience by filling in specific questionnaires, when foreseen by the registry design (Fig. 2). The molecular diagnosis reported for DMD and SMA registries is based on the Human Genome Variation Society nomenclature [26] and is validated by the Curator. The disease terminology used for the clinician-driven registries is based on the Online Mendelian Inheritance in Man (OMIM) codes [27] and the clinical classification on the Orphanet nomenclature [28].

As specifically detailed below for each registry, all clinical protocols of data collection derive from international consensus or include standardised disease-specific datasets (Table 1). Therefore, all registries can share metadata and are potentially interoperable with the respective international efforts.

Patient-reported forms are written in Italian, and their contents match to what implemented in different languages by the other registries participating in the TREAT-NMD Global Registry [24, 25]. The referring POs provide support to patients that need additional information or assistance through their call centers. The clinician-reported forms of dual registries are written directly in English, as they are filled in by physicians who are experts in the field and received ad hoc training for standardisation of this activity*.* Patients are informed about the items that will be collected during the consent talk and the clinical visit.

#### DMD and SMA registries

The DMD and SMA registries collect essential clinical information with mandatory and optional items on clinical status. The datasets were initially defined under the TREAT-NMD project and have recently been revised to take into account updates in standards of care and map novel treatments.

The DMD database collects mainly information on young adults with DMD and Becker muscular dystrophy (overall 161 individuals, mean age 26 years, range of age 4–79 years). The SMA database currently collects information of 560 patients with SMA types 1–4 (mean age 26 years, range of age 0–80 years).

Being part of the TREAT-NMD Global Registry, the two databases have been consulted several times to share aggregated data for feasibility enquires by pharmaceutical companies, inform suitable patients about upcoming trials and contribute information for analyses performed by the TREAT-NMD coordination team [24, 25]. The SMA registry has also been queried for a survey on respiratory care in Italy developed by the Italian SMA POs together with clinical experts [29]. Moreover, the Curator informed families of children with SMA1 when the Expanded Access Program supported by Biogen Idec of the new therapeutic product Spinraza became available [30, 31].

#### CMT registry

This registry is part of the CMT International Database (CMT-ID), which is based on national independent registries and is linked to the Inherited Neuropathy Consortium (INC), a network of 22 centres from US, UK, Italy, Belgium and Australia collecting data for a natural history study on CMT, with a centralised website based at the University of South Florida [32]. The forms derive from the consensus reached at the European NeuroMuscular Centre (ENMC) meeting held in 2009 [33]. Several other countries contribute to the CMT-ID, including South Korea, Brazil, Australia and New Zealand. Although the data of the Italian database are not directly transferred into the CMT-ID, the Italian CMT registry is potentially interoperable with the international CMT-ID. The Scientific coordinator is member of the INC and of the CMT-ID International Oversight Committee, and an ADR representative has recently joined the associated Patient Advocacy Group [34].

The Italian CMT registry is a dual registry, as CMT comprehends a heterogeneous group of disorders with a complex classification and reporting of clinical, electrophysiological and genetic information needs to be accomplished by the attending clinician (Fig. 2). The minimal dataset contains important clinical information about: disease onset; sensory, motor and skeletal manifestations; ability to walk and use of upper limbs; additional features such as optic atrophy and hearing loss; surgical intervention for foot deformities and other skeletal abnormalities; need for shoe inserts, ankle foot orthoses and other devices; electrophysiology; nerve biopsy availability; precise diagnosis, DNA testing results [33]. In addition, clinicians administer the CMT Neuropathy Score/Examination Score version 2 (CMTNS/CMTES) [35] for adults, or the CMT Pediatric Score (CMT PedS) scale [36] for children. These are internationally validated scales and evaluate disability and impairment. Aims of the Registry are to collect information about: 1) epidemiology of CMT in Italy regarding the relative distribution of CMT subtypes, and the mutation frequency for the main CMT genes; 2) natural history course for the main CMT subtypes, as patients are asked to be evaluated every year. Important objectives are also to facilitate recruitment for forthcoming clinical trials and to assist the national and international networks of experts in the development of standard of diagnosis and care.

By June 2018, 721 CMT patients (mean age 46 years, range 5–88 years) had registered and chosen one of the nine reference centres; up to now clinical information has been entered in the Registry for 584 of them.

Registered patients had also the chance to participate in a linked study that required to fill in online self-reported questionnaires related to five important domains: disease course and complications during pregnancy; use, efficacy and tolerability of orthotics and assistive devices; outcome of surgery for skeletal deformities; safety of anaesthesia; occurrence of sleep disorders (including evaluation of fatigue, anxiety and depression). Control individuals also filled in online the questionnaires for pregnancy, anaesthesia, and sleep, and were recruited among friends and unaffected relatives of CMT participants, matched as much as possible for age and sex. By the end of the study (30 November 2017), 306 patients (161 females) and 66 relatives/friends (as healthy controls) had filled in the questionnaires. A huge amount of data have been collected, which will be very important for improving knowledge and giving advice to patients about pregnancy, orthotics, surgery, anaesthesia, sleep and fatigue in CMT, as well as for defining needs, disease burden and standards of care in CMT. Data analysis is under way.

#### SBMA registry

The registry capitalized on the CMT experience for its development. International experts reached an agreement on the items to be collected during the 210th ENMC workshop [12, 37]. Like the CMT Registry, this is a dual registry. The plan is to register about 200 patients, implementing the network of Italian referral centres for SBMA and extending the collaboration to foreign centres of the SBMA European Consortium. The registry is designed to become the basis for an International Registry, being ready for other country to join it. Main scopes are: to test novel outcome measures such as the SBMA Functional Rating Scale (SBMAFRS) [38, 39] and (in selected cases) the quantitative muscle Magnetic Resonance Imaging (MRI) [40]; to perform a natural history study; to build and maintain an SBMA biorepository with collection of biological material; to help defining epidemiology of SBMA in Italy (all main centres that follow these patients are engaged in the registry); to prepare the pathway to clinical trials in Italy. The minimal dataset collects a series of information on comorbidities, current medication, participation to clinical trials, genetic data, family history, age at onset of symptoms, age of loss of fundamental activities of daily life milestones, a complete clinical evaluation, gynecomastia, erectile dysfunction, SBMAFRS, nerve conduction studies/electromyography, availability of biological material. At June 2018, 119 male patients have already registered (mean age 59 years, range 30–81 years), and clinical information has been entered for 91 of them.

#### MGSD registry

The purpose of this registry is to collect uniform clinical and laboratory data of patients with MGSD. The Italian Registry is aligned with the international criteria on the molecular and clinical classification of individuals with MGSD [41, 42]. In fact, patients inclusion need to be sustained by appropriate clinical and laboratory clues as: 1) clinical features observed in the two main forms, namely a) “dynamic form” with exercise intolerance, contractures, hyperCKemia, rhabdomyolisis, myalgia, myoglobinuria and transient weakness and b) “fixed form” with progressive muscle weakness; 2) functional tests (forearm or exercise tests to evaluate lactic acid levels); 3) neurophysiological examinations, 4) neuroimaging features, 5) morphological aspects mainly from muscle biopsy (i.e., presence of vacuoles and/or glycogen accumulation); 6) biochemical findings (residual enzyme activity causing deficiency of glycolytic or glycogenolytic enzymes); 7) molecular genetic changes in the related genes.

By June 2018, 260 individuals (mean age 45 years, range 2–88 years) have been registered. Preliminary results of the clinical observations were disseminated in Italian (AIM; Italian Society of Neurology) and European (European Academy of Neurology) congresses by the Coordinator and some of the other clinicians involved in the registry.

#### TTR-FAP registry

The TTR-FAP Registry has been planned by tertiary clinical centres for peripheral neuropathies, most of which are also members of the Italian CMT network and reference centres for acquired and genetic amyloidosis according to the guidelines of the already active French TTR-FAP Network CORNAMYL [43, 44]. The rationale for developing this registry was based on preliminary results obtained by the Scientific Coordinator and partners indicating that Italy has a peculiar TTR variants geographical distribution with less than one fourth of the patients carrying the common Val30Met mutation with early and late onset cases [44–46]. The only Italian epidemiological investigation of TTR-FAP highlighted a prevalence of 8.8/1,000,000 in Sicily, suggesting that Italy may be an endemic area and favouring the creation of the national registry [46].

This is a dual registry where patients register themselves and choose the centre where they want to be evaluated for the data collection. A collaborating academic centre was also included in the working group for the specific expertise on the psycho-social burden of patients with chronic diseases and their caregivers. Main aims are: to improve understanding of genotype-phenotype relationships, differences in disease presentation, diagnosis and course including inter- and intra-mutation variability; to identify most sensitive outcome measures in a one-year period; to collect information about quality of life, standards of care, psychosocial burden, professional support; to compare the Italian data with other European countries. The minimal dataset collects a series of information on demography, comorbidities, current medication, participation to clinical trials, genetic data, family history, symptoms at onset. The clinical data include neurological, autonomic and cardiologic evaluation, and neurophysiology, echocardiography, cardiac magnetic resonance imaging and scintigraphy. Asymptomatic carriers of TTR mutations are also enrolled in the registry.

At June 2018, 206 individuals have registered (148 symptomatic patients, mean age 62 years, range 29–90 years; 58 asymptomatic carriers, mean age 61 years, range 31–73 years). Other four Italian centres have recently asked to be involved in the registry and have obtained ethical approval by their respective IRBs.

## Discussion

There are several reasons that usually delay the recognition of these rare disorders: a) rarity of those conditions, 2) lack of sufficient awareness of signs and symptoms, 3) adequate knowledge about natural history and diagnostic aspects, 4) rarity of specific treatments, 5) scarcity of clinical follow-up procedures and of standards of care. A registry could provide a powerful database to generate such evidence-based information about clinical and pathophysiological features and supplies also information about current treatments. As a consequence, clinicians and patients expectations are based on implementing the disease-specific registry to allow researchers to be in the best position to examine natural history and different either genotypes or phenotypes of a larger number of these patients.

The Italian NMD Registry was developed to help fulfil these needs of the Italian NMD patient community, which is a major stakeholder of Telethon since its start.

The Registry is based on an IT platform hosting several NMD disease-specific databases, each of them having peculiarities in terms of modality of data collection (patient- or clinician-reported forms) and purposes. The management framework has been periodically revisited to fulfil the upcoming needs. Overall this initiative has been fruitful and relevant from several points of view.

### Patient empowerment

The development of TREAT-NMD-affiliated registries in Italy occurred with the direct engagement of POs with specific interest in muscular dystrophies and motor neuron diseases. Several POs not only expressed the willingness in taking a direct responsibility in the management of these registries, but also decided to work in partnership in order to share tools and knowledge.

Establishing and maintaining registries is a complex and intense effort. The POs and Telethon were aware of it, and through the foundation of ADR, they created a legal entity to make sure that all legal and ethical aspects on patient data stewardship were in place. Safety, protection and wellbeing of participants were key values since the NMD Registry’s start, together with transparency of the process. The POs wished to build up a structure that could guarantee further expansion to incorporate new NMD registries. Accordingly, they designed a simple and flexible governance model, in order to be inclusive and admit any Italian POs with specific interest in the NMD registries.

In 2012, Eurordis, Nord and Cord (the European, American and Canadian Federations of rare disease organizations, respectively) issued a joint declaration underlying the importance of disease registries and inviting collaborative approaches among all stakeholders [17]. Efforts to promote registries’ harmonisation and implementation of standard procedures were taken on by further EC projects, such as EpiRare [47] and RD-Connect [48]. Furthermore, the Federations advocated the importance for a direct involvement of patients both in reporting data and in the registries’ governance, and, more in general, in the creation of partnerships with health professionals and industry.

ADR defined its legal structure in 2009 based on similar principles. A privacy by design model was chosen since the beginning and strong measures were put in place to ensure security and privacy of data collection, maintenance and use. This governance experience allowed the POs to increase their empowerment and gain significant understanding in the highest ethical and legal codes for data management. Moreover, it created a virtuous circle for the empowerment of new POs and their participation in data stewardship and decision-making processes. Several other examples in the NMD field show this positive trend [49–51]. Moreover, these principles are also emphasized in the recent AHQR White Paper “Patient- or Participant-Generated Registries” [52] and in the recent Brocher Foundation workshop report [53].

### The FAIR concept

The Italian NMD Registry mainly satisfies the principles for data management and stewardship envisaged by Eurordis-Nord-Cord [17], European Union Committee of Experts on Rare Diseases (EUCERD) Core Recommendations [18] and RD-Connect, an EU global infrastructure project to support collection of -omics data, personalised medicine and biomarker research on rare diseases [54–56]. In particular, RD-Connect has adopted the guiding principles for data management and stewardship that recommend data be collected in a standardized way and made Findable, Accessible, Interoperable and Reusable (FAIR) [48, 57]. The Italian NMD Registry is findable through the RD-Connect Registry & Biobank Finder [48]. Moreover, the most established registries (DMD, SMA, and CMT registries) are already part of global efforts (TREAT-NMD Global registry in the case of the first two and CMT-ID for the third) [4, 24, 25, 32] and are interoperable with the respective international initiatives. The three most recent ones directly derive by (the SBMA registry) or took inspiration from (the TTR-FAP and GMSD) international consensus efforts (Table 1), thus ensuring even in these cases a certain level of international interoperability with the respective clinical networks. Being the POs involved in the Registry governance, the fulfilment of the patients’ willingness to make their clinical data available to the broad scientific community is guaranteed.

Although the most recent registries (MGSD, SBMA, and TTR-FAP) started out as observational studies (Fig. 3), they were designed as long lasting registries and their protocols included the possibility to reuse data and make them available to third parties. These principles were apprehended also in the patient Informed Consents, thus providing the correct ethical and legal framework for their natural transition from the early stage of clinical studies to the full implementation as registries.

### The ADR - NMD Alliance agreement

ADR and the NMD Alliance acknowledged the common interest to promote collection and management of clinical data of people with NMD. The signed Agreement is a milestone achievement for several reasons. First, it reports in a transparent way the roles and contributions of each stakeholder involved in the NMD Registry and describes the general principles and policies that regulate data management and access. Making these aspects clear and well documented is an essential step to increase the chances of success of a registry [15].

Moreover, the Agreement states that proponent of a new registry, in order to be deployed in the NMD Registry platform, has to provide a rigorous protocol approved by the pertinent IRBs, a business plan and adequate funds. Also, alignment of data collection to the international FAIR principles is strongly recommended and is part of the evaluation process. These rules are particularly reassuring for the ADR, as they imply proper peer-review validation of the scientific content and objectives. This was already the case for the current registries, in particular for the most recent ones (MGSD, SBMA, and TTR-FAP), which are based on clinical study protocols that underwent a rigorous peered-review competition process.

Another important asset of the Agreement is its inclusiveness, a concept that applies to all stakeholders. It concerns any individuals with a NMD condition who can sign up to provide their data for the registry, regardless their membership to any specific POs, as well as any POs with interest in a specific NMD that want to participate in the governance. It also refers to any clinical centre that is willing to contribute to data collection if the scientific quality criteria are satisfied. All involved stakeholders consider this a very important principle and, regardless of who is the first driver for the development of a new registry (either a PO/group of interest, or professionals’ network) this approach will guarantee a large participation of all people who may have specific interest.

Finally, with this document the relevant scientific societies endorse the NMD Registry platform, thus promoting its use within the clinical network and contributing to the dissemination of its governance principles and SOPs. The NMD Registry platform is expected to become the referring structure for future registry initiatives, engaging the young generation of clinicians in data collection through an educational process that has its foundation in the FAIR concepts and ontologies-based classification [48, 57–59]. In fact, the IT platform is working as an intranet structure, allowing the professionals of all centres to share data and information in a secure way, according to the legal requirements and data management good practices. In the future, this potential can be further expanded by implementing new functionalities that may be useful for the clinical network and the POs.

### Evaluation analysis – Risks, challenges, and opportunities

Registries based on voluntary patients’ expression of interest have several advantages, as they are powerful tools to directly involve individuals with a specific condition in the process and grasp their daily life experience. On the other hand, the epidemiological validity of these registries may be limited, as they cannot reasonably map nationwide all existing individuals with that specific conditions [15]; for this type of analysis one should refer to the National Healthcare resources. However, healthcare anonymised data are not easily accessible and may not be suitable to answer precise scientific questions relevant for a certain disease. In addition, individuals that provide their own data for the healthcare databases have a passive role and do not take part in any decision-making process.

Condition-specific registries may have different design and collect patient-reported or clinician-reported forms. Both registration modes have strengths and limitations that must be considered to make sure that the chosen model generates valid knowledge and fulfil all stakeholders’ expectations. Potential risks that may affect the quality of both type of registries concern population bias, data quality and validation, and loss to follow up.

#### Patient-driven registries

A bias in the population selection may derive from a different exposure of patients and families to communication regarding the registry existence and scopes. Those who are regularly followed by centre of expertise or are members of the POs of interest are in general better informed. Moreover, given that POs typically direct families and patients towards their referring experts where possible, it is reasonable to expect that these patients are also followed according to the most updated standards of care, thus affecting the information on adopted standards of care that can be inferred from the registry.

Collecting medical information directly from patients has a number of advantages. First, it captures the direct experience of individuals with a specific condition, it offers a fresh perspective on the quality of life and needs, and engages the family in a responsible way, favouring empowerment and participation. Importantly, it can help reducing the burden on clinicians regarding the workload of data entry. This type of collection proved its usefulness to design clinical trials and helped patient recruitment into trials [6, 24, 25, 58] or, at least, promoted the identification of those that fulfilled the main criteria for a first screening (a clinical trial may have additional inclusion/exclusion criteria that are not identifiable through the registry). Nevertheless, the validity of patient-reported data is exposed to several risks that cannot be underestimated. First of all, the different participants may not uniformly record data and diagnoses. Although clinicians should not revise data inputted by patients [15], at least their validation of the genetic and clinical diagnosis should be ensured [60]. To this regard, if clinical data are required for purposes such as accurate natural history or post-marketing validation of a treatment, clinician-driven registries should be preferred. Another issue concerns patient retention. Filling in the registry forms and keeping data updated may not be a priority for affected individuals and their families or caregivers, especially when the health status of the patient worsens and the disease burden becomes high. Automatic reminders can favour the update of forms, but the support of the referring physician or the registry Curator can be very relevant to guarantee that the registry’s content is correctly updated.

#### Clinician-driven registries

Dual Registries have ambitious aims, which include defining a well-characterized and properly diagnosed patient population, not only for recruitment in clinical trials, but also for natural history studies, validation of outcome measures, creation of biorepositories, epidemiological analyses (if feasible) or surveys. They guarantee both direct input of patients in the registry and the collection of accurate and validated clinical data that are recorded in a systematic and scientifically sound manner and can be rapidly analysed. Of course, to fulfil the above scopes, data need to be validated and of high quality. In our experience, the organisation of training sessions on the functional scales used to record patients’ performance, the implementation of standards of care by the involved centers, as well as the regular monitoring and solicitation of data entry by the registry’s Curator, contribute to implement the databases with data of good quality. These registries may have limitations too, which depend on the nature of the registry itself and, for instance, on the rarity of the diseases and their related clinical and molecular heterogeneity. There may be enrolment biases due to several reasons. One, again, relates to the fact that registration is on a voluntary basis. Participation in the registry is often stimulated by POs or Patient Advocacy Groups linked to clinical study networks and clinicians; therefore, patients are more likely to register if they are already engaged in the patients’ community, and have instruments and contacts to become aware of the registry. In other cases, registration depends on clinical and genetic characteristics; the most severely affected patients are more likely to attend tertiary centers, which in turn are more often involved in registries. Clinicians also may be more prone to encourage registration of patients with genetically defined disorders as compared with those without a precise genetic diagnosis. Upcoming treatments specific for certain disease subgroups may also be a reason for asymmetrical recruitment. Therefore, how much the registry population corresponds to the real world, and how valid the epidemiological inferences are remain open questions. To address and overcome such selection biases is rather difficult. One way is to try to enlarge the registration pathways as much as possible, by clearly defining and disseminating the objectives through the involvement of clinicians in promoting the registry, dedicating time to carefully explain the Informed Consent document, obtaining the agreement and endorsement from the scientific societies, developing the Patient Advocacy Groups’ role and the potentialities of the web. When the registry concerns ultra-rare patients, such as in the case of the MGSD, registration supported by the clinicians themselves may be more effective than waiting for spontaneous registration by affected individuals or their families.

Mapping individuals living with an ultra-rare NMD condition may be even more effective when this occurs directly through an international registry [6, 61, 62]. This could be the case also for the SBMA registry and discussion is ongoing to verify how this can be achieved [12]. In this case, the Italian registry hosted on the NMD registry platform could become an important reference model. Opening the platform to international direct interaction may imply also that the registry structure is fully interoperable. Continuing efforts are ongoing to adopt common data elements and implement ontologies such as the Human Phenome Ontology [59] in line with the international standards and guidelines [58].

Theoretically, registries should define: how many patients they plan to recruit based on a sample size estimation in relation to the aims; how frequently patients should be re-assessed; for how long the registry should last; which statistical analyses will be applied. A registry’s steering committee should include statisticians to deal with such aspects. Plans should be in place for recruiting a reasonable number of patients in a predetermined timeframe for the purposes of the registry, and a statistical analysis plan should be defined right in the registry project design [15].

The aims of the registries currently on the NMD platform are mainly of a descriptive nature and did not require preliminary sample size estimations per se. However, the Steering Committees that planned each database assessed their feasibility by careful evaluation of the expected number of individuals that the national registry could likely map, depending on the disease rarity and the patients’ cohorts of each participating center. The design of the registries was therefore based also on this knowledge, in addition to the international consensus on standardised items.

A very critical point that affects the timeframe and population size that allows the registry reaching statistical significance is its funding. This is particularly true when data collection puts a high burden on clinicians, because data items are numerous and/or patient population is relatively wide. In some cases, registries started as observational studies, with the declared intention of long-term maintenance, update and reuse of data. However, when the initial funding support ends it becomes more and more difficult for clinicians to keep the pace with data recording and updates. In order to overcome this problem, clinicians are encouraged to include fund request for structural sustainability and dedicated personnel in any grant application that entails using the registry as a tool.

Finally, a current limitation of the NMD registry platform is that, being owned by a private entity, it cannot merge data with public healthcare databases. This is clearly an obstacle when one considers the clinical burden in managing medical data and the missed opportunity to share individual biomedical information, which could already be available and complementary to the clinical data collected by the registry. All legally feasible efforts should be put in place to verify the possibilities of matching data with other systems, such as the regional or national healthcare registries [15] or the new NMD European Reference Network initiative [63].

### A private-public agreement

A burning issue raised by EUCERD and the European Medicines Agency (EMA) in 2011 was the need to organise registries by disease and no longer by product [16]. In their view, this would “allow the assessment of patients, not drugs”, avoid fragmentation and duplication of efforts, and promote open access to data as much as possible. They envisaged that partnerships between companies, academia and PO should be fostered, based on transparent rules of governance, with guarantee of high standards of quality and long-term sustainability. Such partnerships should be set in place even before marketing authorization of investigated therapies, and include the possibility to make meaningful comparisons versus natural history or other treatments [16]. These principles have been confirmed also by EBE-EuropaBio [19] and recent EMA [21, 64, 65] and SMA [66] workshops. This view is substantiated by scientific reports that describe the usefulness of well-designed and well-managed rare disease registries as collectors of natural history and “real world” data [67–71]. Few interesting examples of collaborative partnerships engaging patients, health professionals and industry concern international efforts on other diseases, such as the Cystic Fibrosis registry [72, 73] or the Global atypical Haemolytic Uremic Syndrome registry [74]. The Italian NMD Registry represents an interesting example of the interaction between a private entity (the ADR) and academic hospitals or other private or public health institutes to implement the collection of natural history and “real world” clinical data through a nationwide collaborative effort derived from the fact that therapeutic strategies for some NMD were already available or upcoming.

The clinician-driven registries of the Italian NMD Registry platform have not reached a sufficient number of registered subjects yet, or do not have enough follow up to be meaningful for purposes that may be of immediate interest for industry (i.e. for feasibility enquires or long-term natural history) (Fig. 3). These reasons, and the fact that in some cases therapeutic perspectives are not so close, make it difficult to attract the interest of companies on the registries. Ideally, companies with interest in NMD clinical data could concur to a conjunct support, providing a basic contribution acknowledged in a transparent way. This investment would not have any immediate direct return of data on each individual company; however, it would greatly favour boosting data collection, which has the limiting factor of personnel availability at the clinical centres, and contribute to guarantee sustainability for long-term data collection. Each company could then apply to the governing board for specific enquiries once the registry reaches validation for such queries. The ADR is currently developing a sustainability model based on these principles.

A recent paper [75] illustrates the results of a retrospective analysis on product registries conducted by expert pharmaco-epidemiologists. This interesting analysis highlights the delays in getting new registries up and running, the hurdle of patient accrual rate, the biases and missed expectations that affect the outcome of the data collection performed to support post-authorisation studies. The authors suggest possible factors that affect the registry’s performance, which may in part depend upon the characteristics of the registry itself, and indicate the need to clarify registry definitions and the epidemiological concepts, in order to address in a rigorous way the possible biases and increase registries’ effectiveness. In the end, they underline the preference of regulators for patient registries, versus product registries; they also suggest that supporting existing patient registries may improve the timeline of data collection in the post-marketing settings [75]. Although the databases of the NMD registry platform have currently different purposes with respect to the mandatory post-marketing registries therein described, the experience of the NMD Registry and the problems herein highlighted confirm the need for a careful management and for a private-public alliance for long-term sustainability that allows overcoming them.

## Conclusions

The experience of the NMD Registry highlights the importance of the partnership between patients groups and clinicians to facilitate clinical research. It expands the definition of patient-driven registries: not only patients contribute to data collection, but also towards governance. On the one hand, the data stewardship model favoured PO empowerment and direct participation in decision-making processes and provided the clinical network with a tool to collect prospective data in a safe and inclusive manner. On the other hand, it engaged expert clinicians and promoted training on data management and data sharing concepts according to the best clinical practices.

Overall, the databases collect information on more than 2000 individuals with rare or ultra-rare NMDs. The NMD registry platform, however, has the potential to grow and accommodate other registries, not only based on other disease conditions or geographical extension, but also having different research purposes, including capturing real word and post-marketing efficacy data on new treatments that become available for these patient populations.

### Working groups represented by authors

(Details about the affiliation of all participants are reported in the Additional file 1)

*ADR - Executive Board:* Anna Ambrosini (Fondazione Telethon, author); Daniela Lauro (Famiglie SMA); Renato Pocaterra (AISLA); Marco Rasconi (UILDM, current President); Federico Tiberio (ACMT Rete); Salvatore del Vecchio (ASAMSI).

*ADR - ELAC:* Francesco Maria Avato (Ferrara, author); Sara Casati (Milan); Alessandro Martini (Padua); Deborah Mascalzoni (Bolzano); Livio Tronconi (Pavia).

*NMD Alliance Executive Board:* Anna Ambrosini, Lucia Monaco, and Davide Pareyson (Milan, authors); Guido Cavaletti (Monza, author); Maurizio Moggio (Milan); Tiziana Mongini (Turin); Angelo Schenone (Genoa); Gabriele Siciliano (Pisa); Maria Letizia Solinas (Livorno).

*DMD and SMA Registries:* Maria Carmela Pera (Rome, author); Eugenio Maria Mercuri (Rome).

*CMT Registry:* Davide Pareyson and Daniela Calabrese (Milan, authors); Isabella Moroni, Emanuela Pagliano, Chiara Pisciotta, Giuseppe Piscosquito, and Stefano Carlo Previtali (Milan); Franco Gemignani and Isabella Allegri (Parma); Gian Maria Fabrizi and Tiziana Cavallaro (Verona); Angelo Schenone, Marina Grandis, and Chiara Gemelli (Genoa); Luca Padua and Costanza Pazzaglia (Rome); Lucio Santoro and Fiore Manganelli (Naples); Aldo Quattrone and Paola Valentino (Catanzaro); Giuseppe Vita and Anna Mazzeo (Messina).

*MGSD Registry:* Antonio Toscano (Messina, author); Corrado Angelini (Venice); Bruno Bembi (Udine); Andrea Martinuzzi (Conegliano); Paola Tonin (Verona); Massimiliano Filosto (Brescia); Lorenzo Maggi (Milan); Tiziana Mongini (Turin); Claudio Bruno (Genoa); Maria Alice Donati (Florence); Gabriele Siciliano (Pisa); Serenella Servidei (Rome).

*SBMA Registry:* Davide Pareyson and Daniela Calabrese (Milan, authors); Caterina Mariotti, Cinzia Gellera, and Silvia Fenu (Milan); Gianni Sorarù and Giorgia Querin (Padua); Mario Sabatelli and Amelia Conte (Rome).

*TTR-FAP Registry:* Giuseppe Vita (Messina, author); Gian Maria Fabrizi and Tiziana Cavallaro (Verona); Davide Pareyson and Silvia Fenu (Milan); Giampaolo Merlini and Laura Obici (Pavia); Alessandro Mauro (Turin); Marina Grandis and Chiara Gemelli (Genoa); Claudio Rapezzi (Bologna); Mario Sabatelli (Rome); Lucio Santoro and Fiore Manganelli (Naples); Lorenza Magliano (Caserta); Costanza Barcellona (Messina).

## Additional file

Additional file 1: Working Groups – affiliations (DOCX 16 kb)

## Abbreviations

ADR: “Associazione Del Registro” (NMD Registry Association)
AHRQ: Agency for Healthcare Research and Quality
AIM: Associazione Italiana di Miologia (Italian Myology Association)
ASNP: Associazione italiana per lo studio del Sistema Nervoso Periferico (Italian Peripheral Nerve Association)
CMT PedS: CMT Pediatric Score
CMT: Charcot-Marie-Tooth disease
CMT-ID: CMT-International Database
CMTNS/CMTES: CMT Neuropathy Score/Examination Score version 2
DMD: Duchenne muscular dystrophy
EC: European Committee
ELAC: Ethics and Legal Advisory Council
EMA: European Medicines Agencies
ENMC: European NeuroMuscular Centre
EUCERD: European Union Committee of Experts on Rare Diseases
FAIR: Findable, Accessible, Interoperable and Reusable
GDRP: General Data Protection Regulation
INC: Inherited Neuropathy Consortium
IT: Informatics Technology
MGSD: muscle glycogen storage disorders
MRI: Magnetic Resonance Imaging
NMD: NeuroMuscular Disorder
OMIM: Online Mendelian Inheritance in Man
PO: Patient Organisation
PRO: Patient Reported Outcome
SBMA: Spinal and Bulbar Muscular Atrophy
SBMAFRS: SBMA Functional Rating Scale
SMA: Spinal Muscular Atrophy
SOP: Standard Operating Procedure
TTR-FAP: Transthyretin Type-Familial Amyloidotic Polyneuropathy
