# Supplementary material for: The Italian neuromuscular registry: a coordinated platform where patient organizations and clinicians collaborate for data collection and multiple usage
Source: Orphanet J Rare Dis. 2018 Oct 4;13:176. doi: 10.1186/s13023-018-0918-z (PMC6172847; doi:10.1186/s13023-018-0918-z)
Supplement: Supplementary file 1 — Working Groups – affiliations (DOCX 16 kb) [file 13023_2018_918_MOESM1_ESM.docx]

**WORKING GROUPS – affiliations**

*ADR - Executive Board:* Anna Ambrosini (Fondazione Telethon, author); Daniela Lauro (Famiglie SMA, Italy); Renato Pocaterra (AISLA, Italy); Marco Rasconi (UILDM, Italy); Federico Tiberio (ACMT Rete, Italy); Salvatore del Vecchio (ASAMSI, Italy)

*ADR - ELAC:* Francesco Maria Avato (author); Sara Casati (Università di Milano-Bicocca, Italy, and Ethical, legal and Social Issues-ELSI board member for the Biobanking and BioMolecular resources Research Infrastructure, Italian node - BBMRI-IT); Alessandro Martini (Azienda Ospedaliera Università di Padova, Italy); Deborah Mascalzoni (Centre for Research Ethics & Bioethics, EURAC, Bolzano, Italy); Livio Tronconi (Fondazione IRCCS Policlinico Mondino, Università di Pavia, Italy)

*NMD Alliance Executive Board:* Anna Ambrosini, Lucia Monaco, and Davide Pareyson (Milan, authors); Guido Cavaletti (Monza, author); Maurizio Moggio (Fondazione IRCCS Ca’ Granda, Ospedale Maggiore Policlinico Milano, Italy); Tiziana Mongini (Ospedale Molinette, Università di Torino, Italy); Angelo Schenone (IRCCS Azioenda Ospedaliera San Martino, Genova, Italy); Gabriele Siciliano (Azienda Ospedaliera Università Pisana, Pisa, Italy); Maria Letizia Solinas (ASAMSI, Livorno, Italy)

*DMD and SMA Registries:* Maria Carmela Pera (Rome, author); Eugenio Maria Mercuri (Centro clinico NEMO, Policlinico Gemelli, Università Cattolica del Sacro Cuore, Roma, Italy)

*CMT Registry:* Davide Pareyson and Daniela Calabrese (Milan, authors); Isabella Moroni, Emanuela Pagliano, Chiara Pisciotta, and Giuseppe Piscosquito (Fondazione IRCCS Istituto Neurologico Carlo Besta, Milano, Italy) and Stefano Carlo Previtali (IRCCS Ospedale San Raffaele, Milano, Italy); Franco Gemignani and Isabella Allegri (Azienda Ospedaliera, Università di Parma, Italy); Gian Maria Fabrizi and Tiziana Cavallaro (Ospedale Borgoroma, Università di Verona, Italy); Angelo Schenone, Marina Grandis, and Chiara Gemelli (IRCCS Azienda Ospedaliera San Martino, Genova, Italy); Luca Padua and Costanza Pazzaglia (Policlinico Gemelli, Università Cattolica del Sacro Cuore, Roma, Italy); Lucio Santoro and Fiore Manganelli (Azienda Ospedaliera Universitaria Federico II, Napoli, Italy); Aldo Quattrone and Paola Valentino (Università Magna Grecia di Catanzaro, Italy); Giuseppe Vita and Anna Mazzeo (Azienda Ospedaliera Policlinico di Messina, Italy)

*GMSD Registry:* Antonio Toscano (Messina, author); Corrado Angelini (IRCCS Ospedale San Camillo, Venezia, Italy); Bruno Bembi (Azienda Ospedaliera Universitaria S. Maria della Misericordia, Udine, Italy); Andrea Martinuzzi (IRCCS Istituto Eugenio Medea, Conegliano, Italy); Paola Tonin (Ospedale Borgoroma, Università di Verona, Italy); Massimiliano Filosto (Azienda Ospedaliera Universitaria, Spedali Civili di Brescia, Italy); Lorenzo Maggi (Fondazione IRCCS Istituto Neurologico Carlo Besta, Milano, Italy); Tiziana Mongini (Ospedale Molinette, Università di Torino, Italy); Claudio Bruno (IRCCS Istituto Gianna Gaslini, Genova, Italy); Maria Alice Donati (Azienda Ospedaliera Universitaria Anna Meyer, Firenze, Italy); Gabriele Siciliano (Azienda Ospedaliera Università Pisana, Pisa, Italy); Serenella Servidei (Policlinico Gemelli, Università Cattolica del Sacro Cuore, Roma, Italy)

*SBMA Registry:* Davide Pareyson and Daniela Calabrese (Milan, authors); Caterina Mariotti, Cinzia Gellera, and Silvia Fenu (Fondazione IRCCS Istituto Neurologico Carlo Besta, Milano, Italy); Gianni Sorarù and Giorgia Querin (Azienda Ospedaliera Universitaria, Padova, Italy); Mario Sabatelli and Amelia Conte (Policlinico Gemelli, Università Cattolica del Sacro Cuore, Roma, Italy)

*TTR-FAP Registry:* Giuseppe Vita (Messina, author); Gian Maria Fabrizi and Tiziana Cavallaro (Ospedale Borgoroma, Università di Verona, Italy); Davide Pareyson and Silvia Fenu (Fondazione IRCCS Istituto Neurologico Carlo Besta, Milano, Italy); Giampaolo Merlini and Laura Obici (IRCCS Policlinico San Matteo, Pavia, Italy); Alessandro Mauro (IRCCS Istituto Auxologico di Torino, Italy); Marina Grandis and Chiara Gemelli (IRCCS Azienda Ospedaliera San Martino, Genova, Italy); Claudio Rapezzi (Azienda Ospedaliero-Universitaria S. Orsola-Malpighi, Bologna, Italy); Mario Sabatelli (Policlinico Gemelli, Università Cattolica del Sacro Cuore, Roma, Italy); Lucio Santoro and Fiore Manganelli (Azienda Ospedaliera Universitaria Federico II, Napoli, Italy); Lorenza Magliano (Università di Caserta, Italy); Costanza Barcellona (Azienda Ospedaliera Policlinico di Messina, Italy).
